# 788 Lower BMIs Are Correlated with Increased Risk of Graft Loss

**DOI:** 10.1093/jbcr/irae036.329

**Published:** 2024-04-17

**Authors:** Vivie Tran, Delaney A Sauers, Destiny Ugwa, Amber Nanni, Rebecca Gabrilska, Alan Pang, John A Griswold

**Affiliations:** Texas Tech University Health Sciences Center School of Medicine, Lubbock, TX; Texas Tech School of Medicine, Lubbock, TX; Texas Tech University Health Sciences Center, Lubbock, TX; Texas Tech University Health Sciences Center School of Medicine, Lubbock, TX; Texas Tech School of Medicine, Lubbock, TX; Texas Tech University Health Sciences Center, Lubbock, TX; Texas Tech University Health Sciences Center School of Medicine, Lubbock, TX; Texas Tech School of Medicine, Lubbock, TX; Texas Tech University Health Sciences Center, Lubbock, TX; Texas Tech University Health Sciences Center School of Medicine, Lubbock, TX; Texas Tech School of Medicine, Lubbock, TX; Texas Tech University Health Sciences Center, Lubbock, TX; Texas Tech University Health Sciences Center School of Medicine, Lubbock, TX; Texas Tech School of Medicine, Lubbock, TX; Texas Tech University Health Sciences Center, Lubbock, TX; Texas Tech University Health Sciences Center School of Medicine, Lubbock, TX; Texas Tech School of Medicine, Lubbock, TX; Texas Tech University Health Sciences Center, Lubbock, TX; Texas Tech University Health Sciences Center School of Medicine, Lubbock, TX; Texas Tech School of Medicine, Lubbock, TX; Texas Tech University Health Sciences Center, Lubbock, TX

## Abstract

**Introduction:**

The relationship between obesity and burn injuries in the United States remains poorly understood. Skin grafts, frequently used for burn treatments, are considered successful when they effectively merge with the damaged tissue, relying heavily on restored blood flow in the affected area. Inadequate graft incorporation, known as "graft loss," is linked to unfavorable health outcomes. Obesity, characterized by a high body mass index (BMI), is a known condition that reduces peripheral blood flow. Our hypothesis posits that burn patients with higher BMIs are likely to experience lower rates of successful grafting.

**Methods:**

We conducted a retrospective analysis based on a hypothesis, using a cohort of 193 burn patients from October 1, 2012, to October 1, 2022. Inclusion criteria encompassed patients who underwent grafting surgery for burns involving a total body surface area (TBSA) greater than 20%, while females who were pregnant or breastfeeding were excluded. All patients were carefully matched for age, comorbidities, and TBSA percentage. Our primary objective was to utilize the information collected to determine whether patients with higher BMIs exhibited significantly increased rates of graft loss. We employed logistic regression analysis to examine the relationship between BMI and various variables, including the number of surgeries, the presence of infections, graft success, and mortality rates.

**Results:**

We accounted for age, comorbidities, and total body surface area (TBSA) percentage in our analysis. The range of BMIs varied from 17.56 (indicating underweight) to 47.26 (indicating morbid obesity), with an average BMI of 29.81 (classifying as overweight). Our findings revealed a notable link between lower BMIs and increased mortality among burn patients (p=0.0107). Furthermore, lower BMIs were significantly connected to patients requiring more than three grafting surgeries (p=0.0094).

**Conclusions:**

Our study indicates that patients with lower BMIs face elevated mortality risks and are prone to needing multiple grafting surgeries for successful outcomes. This could stem from the fact that lower BMIs might leave the body malnourished and depleted, impacting overall healing. However, it's crucial to note that our study has limitations, including a relatively small sample size and limited geographical diversity.

**Applicability of Research to Practice:**

Medical practitioners can assess surgical risks using BMI, especially for grafting surgeries. Patients with lower BMIs may be considered high-risk and require close post-surgical monitoring. Preoperative nutritional assessments and interventions, such as dietary changes and supplements, can optimize their nutritional status. After surgery, specialized postoperative care, like enhanced nutritional support, can aid healing. Prompt identification and management of signs of malnutrition can enhance recovery.

